# Closed genome sequences of 39 Shiga toxin-producing *Escherichia coli* strains isolated from healthy cattle in Okinawa, Japan

**DOI:** 10.1128/mra.00816-25

**Published:** 2025-09-30

**Authors:** Tetsuya Kakita, Sunao Iyoda, Haruno Taira, Tsuyoshi Kudeken, Yukihiro Akeda, Makoto Ohnishi, Keiji Nakamura

**Affiliations:** 1 Research Center for Infectious Disease, Okinawa Prefectural Institute of Health and Environment, Okinawa, Japan; 2 National Institute of Infectious Diseases, Japan Institute for Health Security 739298 , Tokyo, Japan; 3 Graduate School of Medical Sciences, Kyushu University 12923, Fukuoka, Japan; Rochester Institute of Technology, Rochester, New York, USA

**Keywords:** *Escherichia coli*, Shiga toxin-producing *E. coli*, genomes, complete genome

## Abstract

Cattle are thought to be the primary reservoir of Shiga toxin (Stx)-producing *Escherichia coli* (STEC), an important intestinal pathogen worldwide. Here, we present the closed genome sequences of 39 STEC strains isolated from healthy cattle in a cohort study conducted on the remote islands of Okinawa Prefecture in Japan.

## ANNOUNCEMENT

Shiga toxin (Stx)-producing *Escherichia coli* (STEC) are important foodborne pathogens that cause not only mild diarrhea but also severe hemorrhagic colitis and life-threatening hemolytic uremic syndrome (1). The major virulence factors of STEC are Stxs: Stx1 and Stx2, both of which include multiple variants (Stx1a, Stx1c–Stx1e, and Stx2a–Stx2o) (2–4). Typical STECs, such as O157:H7, have acquired the locus of enterocyte effacement pathogenicity island. Ruminant animals, especially cattle, are thought to be the major natural reservoir of STEC (5–7). To expand the genomic information resources for STEC, we sequenced 39 STEC strains isolated from healthy cattle (*Bos taurus*). All strains were collected during a cohort study examining the prevalence of STEC in cattle.

Between 2023 and 2024, we collected fecal samples from a total of 230 healthy cattle bred on 64 farms on two remote islands in Okinawa Prefecture. Ethical review and approval were not required for the study of animals in accordance with the local legislation and institutional requirements. After culturing in a liquid medium, we analyzed the *stx* gene in the culture using real-time PCR (8). The *stx*-positive culture was then spread onto an *E. coli* agar medium. After overnight incubation, we analyzed the *stx* genes in each colony by PCR. Through this screening process, we obtained a total of 39 STEC isolates. Genomic DNA was purified using the QIAGEN Genomic-tip, following the manufacturer’s instructions, with no modification. The reagents and incubation conditions used in the isolation and DNA purification steps are shown in Table 1. Sequencing libraries were prepared with the Rapid Sequencing Kit 96 V14 (SQK-RBK114.96; Oxford Nanopore Technologies [ONT]) following the manufacturer’s protocol and sequenced using MinION with R10.4.1 flow cell (ONT). After sequencing, read data in a FASTQ file format were generated using Dorado v.0.8.3 (9) in the Super accurate basecalling mode with the DNA basecalling model dna_r10.4.1_e8.2_400bps_sup@v5.0.0. The raw ONT reads of each strain were assembled using the Hybracter pipeline v.0.11.0 (10) without the subsampling read option. The genomes of strains OkiPb01712 and OkiPb01714 were not closed using the raw reads. Therefore, the low-quality reads were filtered using NanoFilt v.2.8.0 (11) with the following Q (quality) scores: a Q score of over 8 for strain OkiPb01712 and a Q score of over 20 for strain OkiPb01714. The species in each strain were verified using the species prediction option in ECTyper v1.0.0 (12). All bioinformatic tools were run using their default parameters, except for the aforementioned options.

**TABLE 1:** General features, assembly metrics, and accession numbers of 39 STEC genome sequences

	Method^*a*^								Chromosome						Nanopore reads	
Strain	Liquid media	Agar media	Temperature (°C)^*b*^	Genomic- tip	Serotype^*c*^	*stx* subtype^*d*^	*eae^d^*	No. of genomes	Genome size (bp)	GC content (%)	Completeness (%)^*e*^	Coverage (×)	GenBank accession no.	Plasmid size(s) (bp) (GenBank accession no.)	No of reads^*f*^	SRA accession no.
OkiPb01676	BPW	XM-G	42	100/G	O109:H16	2a	−^*g*^	3	5,168,446	50.71	99.6	98	AP042480	186,285 (AP042481); 37,657 (AP042482)	126,923	DRR667157
OkiPb01677	BPW	STEC	37	100/G	O174:H21	2c	−	12	5,300,047	50.74	99.6	173	AP042483	109,222 (AP042484); 98,483 (AP042485); 33,673 (AP042486); 6,754 (AP042487); 5,631 (AP042488); 5,569 (AP042489); 5,114 (AP042490); 4,352 (AP042491); 4,237 (AP042492); 3,064 (AP042493); 1,546 (AP042494);	226,573	DRR667158
OkiPb01678	mEC	XM-G	42	100/G	O104:H7	2a	−	5	5,072,667	50.91	99.9	160	AP042495	122,352 (AP042496); 92,451 (AP042497); 6,774 (AP042498); 5,171 (AP042499)	191,807	DRR667159
OkiPb01679	BPW	STEC	42	100/G	O177:H25	2c	+	6	5,282,706	50.63	100.0	207	AP042500	98,160 (AP042501); 88,796 (AP042502); 50,807 (AP042503); 11,445 (AP042504); 9,875 (AP042505)	224,721	DRR667160
OkiPb01680	mEC	STEC	37	100/G	O156:H25	1a	+	4	5,281,371	50.6	99.9	271	AP042506	61,578 (AP042507); 46,161 (AP042508); 5,171 (AP042509)	218,378	DRR667161
OkiPb01681	BPW	XM-G	37	100/G	O88:H25	1a, 2a	−	2	4,999,534	50.83	99.9	134	AP042510	95,232 (AP042511)	145,600	DRR667162
OkiPb01682	BPW	XM-G	37	100/G	O181:H51	2a	−	3	4,831,654	50.92	99.9	315	AP042512	173,793 (AP042513); 7,769 (AP042514)	258,666	DRR667163
OkiPb01683	mEC	STEC	37	100/G	O156:H25	1a	+	4	5,297,916	50.55	99.9	124	AP042515	230,086 (AP042516); 82,388 (AP042517); 5,167 (AP042518)	125,864	DRR667164
OkiPb01684	BPW	STEC	37	100/G	O109:H10	2a	+	4	5,261,191	50.87	100	269	AP042519	70,980 (AP042520); 5,163 (AP042521); 1,546 (AP042522)	231,868	DRR667165
OkiPb01685	BPW	XM-G	37	100/G	Ountypable:H7	1a	−	6	4,917,321	50.85	99.9	236	AP042523	97,467 (AP042524); 57,999 (AP042525); 7,334 (AP042526); 6,822 (AP042527); 6,754 (AP042528)	226,667	DRR667166
OkiPb01686	mEC	STEC	37	20/G	O157:H7	2c	+	3	5,453,361	50.52	100	207	AP042529	94,160 (AP042530); 6,042 (AP042531)	205,959	DRR667167
OkiPb01687	mEC	STEC	42	20/G	O157:H7	2c	+	3	5,451,560	50.52	99.9	256	AP042532	93,160 (AP042533); 6,042 (AP042534)	201,206	DRR667168
OkiPb01689	mEC	STEC	37	20/G	O111:H8	1a	+	8	5,343,242	50.57	99.9	340	AP042540	122,771 (AP042541); 98,602 (AP042542); 77,952 (AP042543); 6,774 (AP042544); 6,757 (AP042545); 6,645 (AP042546); 5,236 (AP042547)	275,809	DRR667170
OkiPb01690	BPW	XM-G	37	20/G	O109:H16	2a	−	3	5,208,049	50.69	99.9	208	AP042548	186,285 (AP042549); 37,657 (AP042550)	265,276	DRR667171
OkiPb01691	BPW	XM-G	37	20/G	O151/O118:H16	1a	+	3	5,643,813	50.69	100	150	AP042551	88,840 (AP042552); 6,673 (AP042553)	241,142	DRR667172
OkiPb01692	mEC	XM-G	37	20/G	O171:H2	2d	−	7	5,457,332	50.81	99.9	258	AP042554	123,790 (AP042555); 7,286 (AP042556); 5,631 (AP042557); 5,423 (AP042558); 4,232 (AP042559); 3,625 (AP042560)	294,268	DRR667173
OkiPb01693	BPW	STEC	37	20/G	O157:H7	2c	+	3	5,494,199	50.54	100	162	AP042561	82,292 (AP042562); 6,042 (AP042563)	193,763	DRR667174
OkiPb01694	BPW	STEC	42	20/G	O8:H4	1a, 2d	−	6	4,971,397	50.89	100	198	AP042564	142,337 (AP042565); 109,836 (AP042566); 34,026 (AP042567); 7,136 (AP042568); 1,506 (AP042569)	227,380	DRR667175
OkiPb01695	BPW	STEC	37	20/G	Ountypable:H20	2g	−	4	5,026,011	50.75	99.7	131	AP042570	138,561 (AP042571); 71,467 (AP042572); 38,788 (AP042573)	157,429	DRR667176
OkiPb01696	mEC	XM-G	37	20/G	O171:H2	2d	−	9	5,461,333	50.88	99.9	80	AP042574	121,100 (AP042575); 7,286 (AP042576); 7,136 (AP042577); 5,631 (AP042578); 5,463 (AP042579); 3,625 (AP042580); 3,064 (AP042581); 2,255 (AP042582);	107,443	DRR667177
OkiPb01697	mEC	XM-G	37	20/G	O8:H8	2a, 2d	−	2	4,969,035	50.67	99.6	106	AP042583	130,200 (AP042584)	138,068	DRR667178
OkiPb01698	mEC	XM-G	37	20/G	O181:H49	2a	−	6	4,841,896	50.91	99.9	162	AP042585	173,896 (AP042586); 108,543 (AP042587); 58,563 (AP042588); 28,753 (AP042589); 7,769 (AP042590)	207,675	DRR667179
OkiPb01699	mEC	STEC	42	20/G	O157:H7	2c	+	3	5,473,218	50.56	100	129	AP042591	93,159 (AP042592); 6,042 (AP042593)	203,402	DRR667180
OkiPb01700	mEC	STEC	37	20/G	O156:H25	1a	+	4	5,283,251	50.6	99.9	93	AP042594	61,743 (AP042595); 46,161 (AP042596); 5,171 (AP042597)	133,892	DRR667181
OkiPb01701	BPW	STEC	42	20/G	O116:H16	2a	−	7	5,299,229	50.67	99.9	63	AP042598	225,302 (AP042599); 69,930 (AP042600); 45,508 (AP042601); 7,924 (AP042602); 5,114 (AP042603); 4,091 (AP042604)	103,220	DRR667182
OkiPb01702	BPW	STEC	42	20/G	O26:H11	1a	+	11	5,807,644	50.56	99.9	102	AP042605	223,606 (AP042606); 98,420 (AP042607); 92,602 (AP042608); 70,763 (AP042609); 47,469 (AP042610); 6,773 (AP042611); 6,758 (AP042612); 6,675 (AP042613); 3,667 (AP042614); 2,464 (AP042615);	198,394	DRR667183
OkiPb01703	mEC	STEC	37	20/G	O116:H16	2a	−	7	5,300,431	50.67	99.9	167	AP042616	225,300 (AP042617); 69,931 (AP042618); 45,508 (AP042619); 7,925 (AP042620); 5,114 (AP042621); 4,091 (AP042622)	197,694	DRR667184
OkiPb01704	BPW	STEC	37	20/G	O116:H16	2a	−	7	5,299,253	50.67	99.9	71	AP042623	225,296 (AP042624); 69,930 (AP042625); 45,508 (AP042626); 7,924 (AP042627); 5,114 (AP042628); 4,091 (AP042629)	94,830	DRR667185
OkiPb01705	BPW	STEC	37	20/G	O116:H16	2a	−	7	5,300,435	50.67	99.9	136	AP042630	217,593 (AP042631); 69,930 (AP042632); 45,508 (AP042633); 7,925 (AP042634); 5,114 (AP042635); 4,091 (AP042636)	169,754	DRR667186
OkiPb01706	BPW	STEC	42	20/G	O116:H16	2a	−	7	5,300,368	50.67	99.9	254	AP042637	225,298 (AP042638); 69,931 (AP042639); 45,508 (AP042640); 7,925 (AP042641); 5,114 (AP042642); 4,091 (AP042643)	328,682	DRR667187
OkiPb01707	BPW	STEC	37	20/G	O151/O118:H16	1a	+	8	5,688,201	50.7	100	188	AP042644	222,727 (AP042645); 94,992 (AP042646); 88,839 (AP042647); 49,760 (AP042648); 39,727 (AP042649); 6,673 (AP042650); 2,579 (AP042651)	338,908	DRR667188
OkiPb01708	mEC	XM-G	37	20/G	O116:H16	2a	−	7	5,300,443	50.67	99.9	223	AP042652	225,298 (AP042653); 69,931 (AP042654); 45,508 (AP042655); 7,925 (AP042656); 5,114 (AP042657); 4,091 (AP042658)	353,610	DRR667189
OkiPb01709	BPW	XM-G	37	20/G	O116:H16	2a	−	7	5,300,438	50.67	99.9	177	AP042659	225,194 (AP042660); 69,930 (AP042661); 45,508 (AP042662); 7,925 (AP042663); 5,114 (AP042664); 4,091 (AP042665)	268,051	DRR667190
OkiPb01710	BPW	STEC	37	20/G	O116:H16	2a	−	7	5,300,399	50.67	99.9	186	AP042666	225,386 (AP042667); 69,931 (AP042668); 45,508 (AP042669); 7,925 (AP042670); 5,114 (AP042671); 4,091 (AP042672)	190,583	DRR667191
OkiPb01711	BPW	STEC	42	20/G	O84:H2	1a	+	3	5,713,410	50.64	99.9	110	AP042673	65,720 (AP042674); 8,379 (AP042675)	143,911	DRR667192
OkiPb01712	mEC	STEC	37	20/G	O104:H7	2a	−	6	5,168,815	50.86	99.9	85	AP042676	123,668 (AP042677); 122,360 (AP042678); 89,760 (AP042679); 6,774 (AP042680); 5,171 (AP042681)	121,376 (117,252)	DRR667193
OkiPb01713	BPW	STEC	37	20/G	O5:H9	1a × 2	+	5	5,297,972	50.58	99.9	110	AP042682	76,471 (AP042683); 7,323 (AP042684); 2,679 (AP042685); 1,552 (AP042686)	140,653	DRR667194
OkiPb01714	mEC	XM-G	37	20/G	O171:H2	2d	−	8	5,432,029	50.8	99.9	161	AP042687	112,441 (AP042688); 49,999 (AP042689); 7,286 (AP042690); 5,631 (AP042691); 5,444 (AP042692); 3,625 (AP042693); 3,064 (AP042694)	223,391 (152,124)	DRR667195
OkiPb01715	BPW	XM-G	37	20/G	O168:H8	2a	−	6	5,282,311	50.69	99.9	75	AP042695	223,921 (AP042696); 121,500 (AP042697); 97,775 (AP042698); 33,585 (AP042699); 7,925 (AP042700)	222,585	DRR707817

^*a*^ Media, growth condition, and DNA purification kit employed in the isolation and DNA purification steps (BPW, buffered peptone water [Oxoid]; mEC, mEC broth [Eiken]; XM-G, XM-G agar [Nissui]; STEC, CHROMagar STEC [Kanto]).

^*b*^ The incubation temperature for cultivation on the agar medium.

^*c*^ Determined using ECtyper (12).

^*d*^ Determined by BLASTN as previously described (13).

^*e*^ Assessed using CheckM v.1.2.2 (14).

^*f*^ For OkiPb01712 and OkiPB01714, the total number of reads used in the genome assembly is shown in parentheses.

^*g*^ “−” indicates negative and “+” indicates positive.

All the isolates were found to be *E. coli*, with 22 different serotypes, suggesting that various STECs, including typical STECs such as O157:H7, were present in the healthy cattle (Table 1). The main subtypes of Stx found in the cattle isolates were Stx1a, Stx2a, and Stx2c, which have been reported to be frequently detected in major STEC serotypes (1). Table 1 also provides assembly metrics and accession numbers for each genome. The sizes of chromosomes and sequence coverages ranged from 4,831,654 to 5,807,644 bp (average: 5,289,280 bp) and from 63 to 340 (average: 169), respectively. The average G + C content for all 39 chromosomal sequences was 50.7%. Each genome contained between 1 and 11 plasmids.

## Data Availability

The raw read sequences and closed genome sequences generated for this study have been deposited in GenBank/EMBL/DDBJ under the accession numbers listed in Table 1 and the BioProject accession number PRJDB20538.
